# Plant-Produced Antigen Displaying Virus-Like Particles Evokes Potent Antibody Responses against West Nile Virus in Mice

**DOI:** 10.3390/vaccines9010060

**Published:** 2021-01-17

**Authors:** Junyun He, Huafang Lai, Adrian Esqueda, Qiang Chen

**Affiliations:** 1 The Biodesign Institute, Arizona State University, Tempe, AZ 85287, USA; junyunhe0123@gmail.com (J.H.); huafang.lai@asu.edu (H.L.); Adrian.Esqueda@asu.edu (A.E.); 2 School of Life Sciences, Arizona State University, Tempe, AZ 85287, USA

**Keywords:** West Nile virus, virus-like particle (VLP), plant-made vaccine, vaccine, envelope protein, domain III (DIII)

## Abstract

In this study, we developed a hepatitis B core antigen (HBcAg)-based virus-like particle (VLP) that displays the West Nile virus (WNV) Envelope protein domain III (wDIII) as a vaccine candidate for WNV. The HBcAg-wDIII fusion protein was quickly produced in *Nicotiana benthamiana* plants and reached a high expression level of approximately 1.2 mg of fusion protein per gram of leaf fresh weight within six days post gene infiltration. Electron microscopy and gradient centrifugation analysis indicated that the introduction of wDIII did not interfere with VLP formation and HBcAg-wDIII successfully assembled into VLPs. HBcAg-wDIII VLPs can be easily purified in large quantities from *Nicotiana benthamiana* leaves to >95% homogeneity. Further analysis revealed that the wDIII was displayed properly and demonstrated specific binding to an anti-wDIII monoclonal antibody that recognizes a conformational epitope of wDIII. Notably, HBcAg-wDIII VLPs were shown to be highly immunogenic and elicited potent humoral responses in mice with antigen-specific IgG titers equivalent to that of protective wDIII antigens in previous studies. Thus, our wDIII-based VLP vaccine offers an attractive option for developing effective, safe, and low-cost vaccines against WNV.

## 1. Introduction

West Nile virus (WNV) is a positive-stranded, enveloped RNA virus in the *Flavivirus* genus of the *Flaviviridae* family. WNV is neurotropic and can infect the central nervous system of humans and animals. Symptoms of a WNV infection range from fever to neuroinvasive complications with the elderly, immunocompromised individuals, and people with certain genetic factors as the most vulnerable population to develop encephalitis, meningitis, long-term morbidity, and death [1]. Since its arrival in the Western hemisphere in 1999, outbreaks of WNV have become more frequent and severe with a higher instance of patients with neuroinvasive complications [2]. However, currently, there is no approved WNV vaccine or therapeutic treatment approved for human use. This calls for the development of vaccines and production technologies that can quickly bring the vaccines to clinics at low cost.

The WNV Envelope (E) glycoprotein plays an important role in mediating viral binding to cellular receptors and subsequent virus-cell membrane fusion and it is a major target for the host humoral response [3]. Within the typical three-domain architecture of a flavivirus E protein [4], domain III of the WNV E protein (wDIII) contains the majority of type-specific neutralizing epitopes that elicit potent neutralizing antibody responses and/or protective immunity [5]. Neutralizing antibody responses has been shown to play an important role in the protection against infection of flaviviruses [6]. For example, a neutralizing antibody response has been found to correlate with protection for approved human vaccines against tick-borne encephalitis virus and yellow fever virus [7,8]. As a result, wDIII has been expressed in insect and bacterial cell cultures [9,10], as well as in plants [11], and tested as a WNV vaccine candidate. However, the development of these wDIII-based vaccines has encountered difficulties in producing soluble wDIII with native epitopes, sufficient yield, or scalability to support commercial development [10,11]. 

In response, we generated a wDIII-based subunit vaccine in the form of wDIII-displaying virus-like particles (VLPs) based on the hepatitis B core antigen (HBcAg). We selected this protein-based yet particulate vaccine candidate and opted to produce it with a plant-based transient expression system in order to increase the yield, stability, immunogenicity, and cost-effectiveness of a wDIII-based vaccine candidate. 

## 2. Materials and Methods

### 2.1. Construction of Plant Expression Vectors of HBcAg-wDIII

The coding sequence of wDIII (amino acid 296-415, Genbank Acc.No. AF196835) was synthesized with optimized *N. benthamiana* codons and was fused to the 3′ of HbcAg as described previously [12]. The DNA construct of HBcAg-wDIII was then cloned into the TMV-based expression vector pIC11599 of the MagnICON system [13]. The MagnICON vectors were chosen because they have been demonstrated to drive high-level accumulation of recombinant proteins in *N. benthamiana* plants [14,15,16,17].

### 2.2. Expression of HBcAg-wDIII in N. benthamiana Leaves

Plant expression vectors were transformed into *Agrobacterium tumefaciens* GV3101 by electroporation as described previously [18] and agroinfiltrated into *N. benthamiana* plants. Specifically, the GV3101 strain containing the HBcAg-wDIII 3’ module (pICH11599-HBcAg-wDIII) was co-infiltrated with the 5’ module (pICH20999) and the integrase construct (pICH14011) as described previously [19,20,21,22]. 

### 2.3. Purification and Characterization of HBcAg-wDIII from N. benthamiana Leaves

Agroinfiltrated *N. benthamiana* leaves were harvested 4–10 days post agroinfiltration (DPI) for evaluating the temporal pattern of HBcAg-wDIII expression. Leaves were harvested 6 DPI for other protein analysis. Leaves were homogenized in extraction buffer (100 mM Tris-HCl, pH 8.0, 150 mM NaCl, 1 mM PMSF, tablet protease inhibitor cocktail (Sigma, Germany) at 1 mL/g leaf fresh weight (LFW)). Plant clarified extract was obtained by centrifugation at 18,000× *g* for 30 min at 4 °C. The pH of the clarified extract was adjusted to 5.0, incubated for 12 h at 4 °C, and then subjected to centrifugation at 18,000× *g* for 30 min at 4 °C. The supernatant was recovered, pH adjusted back to 8.0, and subjected to another round of centrifugation. The supernatant was then subjected to sucrose gradient sedimentation as described previously [23]. Briefly, clarified plant extracts were layered onto 30 mL linear 10–60% sucrose gradients (dissolved in 50 mM Tris-HCl, pH 8.0, 150 mM NaCl) and centrifuged at 280,000 ×  *g* for 3  h at 4 °C. Thirty fractions (1 mL each) were collected and assayed for HBcAg and wDIII content and VLP assembly by ELISA, SDS-PAGE, and electron microscopy. The purity of HBcAg-wDIII was estimated by quantitating Coomassie blue-stained protein bands on SDS-PAGE using a densitometer as described previously [24]. 

### 2.4. SDS-PAGE and Western Blot Analysis

SDS-PAGE and Western blot analysis were performed as described previously [25]. Briefly, protein samples containing HBcAg-wDIII were subjected to 12% SDS-PAGE under reducing (5% *v*/*v* β-mercaptoethanol) conditions. Gels were either used to transfer proteins onto PVDF membranes (MilliporeSigma, Burlington, MA, USA) or stained with Coomassie blue. For Western blot analysis, membranes were first incubated with E16, a humanized mouse mAb against wDIII [24], and then with a goat anti-human kappa antibody conjugated with horseradish peroxidase (HRP) (Southern Biotech, Birmingham, AL, USA). The membranes were then developed by incubating with an “ECL plus” Western blot detection system (Amersham Biosciences, Piscataway, NJ, USA) to detect specific binding. 

### 2.5. ELISAs

The expression of HBcAg-wDIII protein in leaves was measured by a sandwich ELISA. Microtiter plates (Corning Incorporated, Tewksbury, MA, USA) were coated at 1 μg/mL E16 mAb in coating buffer (100 mM Na_2_CO_3_, pH 9.6), washed with PBST (PBS containing 0.1% Tween-20), and blocked with blocking buffer (PBS containing 5% milk). The plates were then incubated with clarified plant extracts. wDIII [11] and plant extracts from leaves that were not infiltrated with the HBcAg-wDIII construct were used as positive and negative controls, respectively. Plates were subsequently incubated with a rabbit anti-WNV DIII polyclonal antibody [26], followed by an HRP-conjugated goat anti-rabbit IgG (Southern Biotech, Birmingham, AL, USA). The plates were developed with 3, 3′, 5, 5′-Tetramethylbenzidine (TMB) substrate (KPL Inc., Milford, MA, USA) and read at 450 nm. 

HBcAg-wDIII in sucrose gradient fractions were also quantitated by the same procedure when levels of the wDIII moiety in the fusion protein were measured. For levels of the HbcAg moiety, an HRP-conjugated anti-HbcAg mAb (Abcam, Cambridge, MA, USA) was used as the detection antibody with purified HbcAg [23] as the reference standard. 

The specific binding of the E16 mAb by HBcAg-wDIII was determined as described previously [24]. Briefly, purified HBcAg-wDIII was coated on ELISA plates and incubated with E16. After washing, an HRP-conjugated goat anti-human-gamma antibody (Southern Biotech, Birmingham, AL, USA) was used to detect bound antibodies. A plant-produced anti-Ebola 6D8 mAb [27] was used as an isotype negative control. 

ELISA was also used to measure the titer of wDIII-specific IgG in mouse sera. Plates were coated with wDIII and blocked with PBS containing 1% bovine serum albumin (BSA). Subsequently, a serial dilution of serum was added to the plates. The plates were then incubated with an HRP-conjugated goat anti-mouse IgG (Southern Biotech, Birmingham, AL, USA) and developed with TMB substrate (KPL Inc., Milford, MA, USA). Geometric mean titer (GMT) was calculated for each group at various time points and used to express the titer of the wDIII-specific IgG for each mouse group.

The ELISA for determining the IgG1 and IgG2a subtypes was performed according to previously published protocols [11] using plates coated with wDIII. Instead of using an anti-mouse total IgG antibody, IgG subtype-specific antibodies were used for detection. Specifically, HRP-conjugated goat anti-mouse IgG1 (Santa Cruz Biotech, Dallas, TX, USA) and anti-mouse IgG2a (Southern Biotech, Birmingham, AL, USA) were used as detection antibodies. 

All ELISA measurements were repeated at least three times independently with each sample in triplicate.

### 2.6. Electron Microscopy

HBcAg-wDIII VLPs from peak sucrose gradient fractions were subjected to negative staining with 0.5% aqueous uranyl acetate and transmission electron microscopy analysis with a Philips CM-12S microscope as described previously [28].

### 2.7. Yeast Surface Display

Yeast cells expressing wDIII were stained with mouse sera or the E16 mAb as described previously [24]. Briefly, yeast cells were grown to log phase and induced for wDIII expression. Pooled mice sera collected in week 11 of the HBcAg-wDIII VLP immunization experiments were diluted with PBS (1:1000) and then incubated with yeast cells. Pooled sera from the PBS mock-immunized mice was used as a negative control. The specific binding of antibodies in immunized mouse sera to wDIII on yeast cells was detected by staining yeast with an Alexa Fluor 488-conjugated goat anti-mouse IgG secondary antibody (Invitrogen, Waltham, MA, USA). For a competitive binding assay, yeast cells were pre-incubated with mouse sera and then incubated with E16, a potent neutralizing humanized IgG1 mAb against wDIII. After washing, yeast cells were stained with an Alexa Fluor 488-conjugated goat anti-human IgG secondary antibody (Invitrogen, Waltham, MA, USA). Yeast cells stained with Alexa Fluor 488-conjugated secondary antibodies were then analyzed on a BD FACSCalibur flow cytometer. 

### 2.8. Mouse Experiments

All animal work was approved by the institutional animal care and use committee (IACUC) (Approval #09-1041R) and performed in accordance with the guidelines of the National Institutes of Health (NIH) for the care and use of laboratory animals. Two groups (*n* = 6 per group) of five-week-old female BALB/C mice were used for the experiment with group 1 receiving saline buffer (PBS) with alum as mock immunized control and group 2 receiving 25 μg of plant-derived HBcAg-wDIII per dosage. On day 0, mice were immunized subcutaneously with 100 μL of material containing saline (group 1) or 25 μg (group 2) purified HBcAg-wDIII protein in PBS with alum as the adjuvant (Sigma, Burlington, MA, USA; wDIII Protein solution: alum volume ratio = 1:1). Immunized animals were boosted three times (on days 21, 42, and 63) with the same dosage and route used for primary immunization. Blood was sampled from the retro-orbital vein on day 0 before the immunization (pre-immune sample) and on days 14 (2 weeks), 35 (5 weeks), 56 (8 weeks), and 77 (11 weeks) after the 1st immunization.

### 2.9. Statistical Analyses

Statistical analyses were performed using GraphPad Prism software version 8.4 (GraphPad, San Diego, CA, USA). Non-linear regression analysis using a one-site binding model was used to determine the Kd of wDIII binding to the E16 mAb. Comparisons of wDIII-specific IgG titers and IgG subtype titers between mouse groups or between samples collected at various time points were performed by t-test. A *p*-value of <0.05 indicated statistically significant differences.

## 3. Results

### 3.1. Expression of HBcAg-wDIII in Nicotiana benthamiana Plants

**Figure 1:**
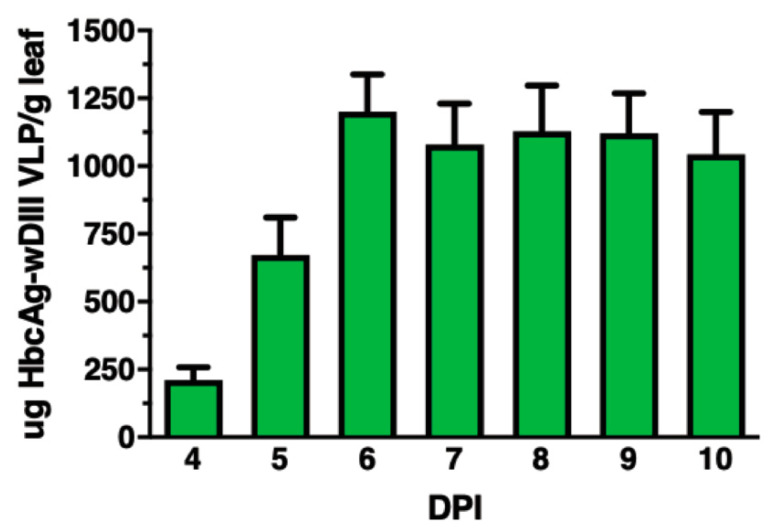
Temporal expression patterns of HBcAg-wDIII plant leaves. Total protein from *N. benthamiana* leaves infiltrated with HBcAg-wDIII construct was extracted on 4–10 DPI and analyzed by an ELISA with the E16 mAb, which recognizes a conformational epitope on wDIII as the capture antibody, and detected with a polyclonal anti-wDIII antibody. The means ± SD of samples from three independent experiments are presented.

**Figure 2:**
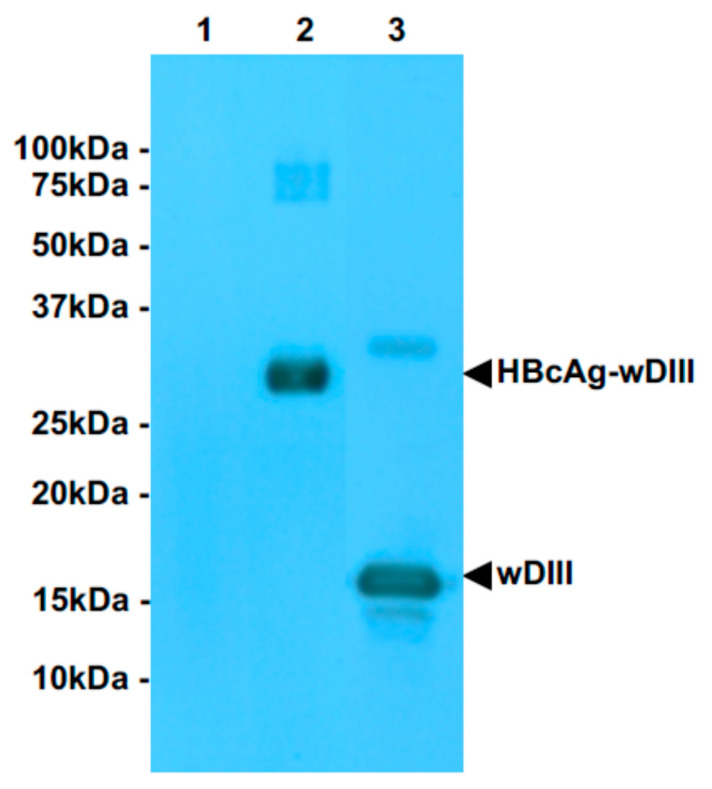
Western blot analysis of HBcAg-wDIII expression in *N. benthamiana*. HBcAg-wDIII was extracted from leaves and separated on SDS-PAGE gels and transferred onto PVDF membranes. wDIII-specific E16 mAb was used to detect HBcAg-wDIII. lane 1: negative control leaf proteins extracted from un-infiltrated leaves; lanes 2: sample collected 6 DPI from leaves agroinfiltrated with HBcAg-wDIII construct; lane 3: *E. coli*-produced wDIII as a positive control.

The wDIII coding sequence was fused to the 3′ end of the gene of HBcAg and cloned into the plant expression vector 11599 for expressing HBcAg-wDIII in the apoplast of plant leaves. The HBcAg-wDIII expression vector was transformed into *A. tumefaciens* and subsequently agroinfiltrated into *N. benthamiana* leaves. An ELISA was used to examine and quantify the expression of HBcAg-wDIII. As shown in Figure 1, HBcAg-wDIII was expressed robustly and reached the highest level of production 6 DPI with an average accumulation of 1200 μg/g LFW (Figure 1). This accumulation level of HBcAg-wDIII represents one of the highest expression levels of recombinant proteins in plants [12]. Western blot analysis was used to confirm the identity of plant-produced HBcAg-wDIII, which detected a positive band with the predicted molecular mass for the HBcAg-wDIII fusion protein (31.7 kDa) (Figure 2, lane 2). The lack of a positive band in the negative control leaf samples (Figure 2, lane 1) confirmed the specificity of the HBcAg-wDIII band. 

### 3.2. VLP Assembly of Plant-Expressed HBcAg-wDIII

**Figure 3:**
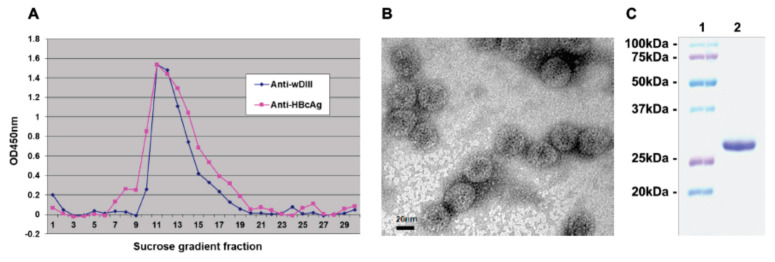
VLP assembly of plant-produced HBcAg-wDIII. Protein extract from HBcAg-wDIII-expressing leaves was subjected to a 10–60% sucrose gradient centrifugation. (**A**) ELISA of sucrose gradient fractions. An antibody against wDIII or HbcAg was used to detect the wDIII or HbcAg moiety in HBcAg-wDIII, respectively. (**B**) Electron microscopy of HBcAg-wDIII from peak fractions of the sucrose gradient. Bar = 20 nm. One representative field is shown. (**C**) SDS-PAGE analysis. Lane 1: molecular weight marker; lane 2: HBcAg-wDIII from peak fractions of the sucrose gradient.

To assess if HBcAg-wDIII was assembled into VLPs, plant protein extracts were subjected to sucrose gradient sedimentation. Analysis of gradient fractions with ELISA showed that HBcAg-wDIII was detected in the same particulate fractions regardless of whether anti-HBcAg or anti-wDIII antibodies were used for detection in ELISA (Figure 3A). This distribution of HBcAg-wDIII overlaps with that of the parent HBcAg VLPs in the sucrose gradient we reported previously [23], indicating the successful VLP assembly. Electron microscopy analysis of the HBcAg-wDIII peak sucrose gradient fractions conclusively confirmed the presence of typical HBcAg-based VLPs with a diameter of ~25–30 nm (Figure 3B). The purity of HBcAg-wDIII VLPs recovered from sucrose gradient fractions was shown to be greater than 95% pure by SDS-PAGE (Figure 3C), indicating that the one-step sucrose gradient centrifugation process efficiently removed most plant host proteins and enriched HBcAg-wDIII to a high degree of homogeneity. 

### 3.3. wDIII Was Displayed Properly by HBcAg VLPs with the Native wDIII Conformation

**Figure 4:**
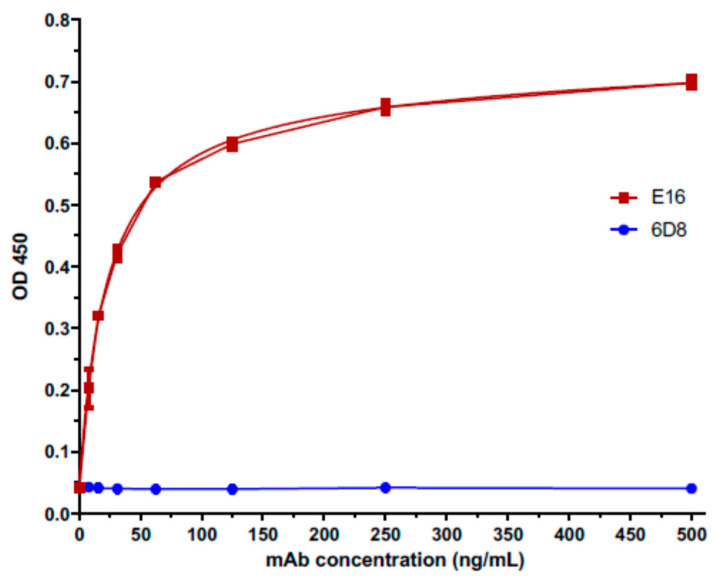
Specific binding of HBcAg-wDIII to a neutralizing mAb (E16) against WNV. Serial dilutions of the E16 mAb were incubated in microtiter plates coated with plant-produced HBcAg-wDIII and detected with an HRP-conjugated anti-human gamma antibody. A plant-produced anti-Ebola mAb (6D8) was used as a negative isotype control. Mean ± SD of samples from three independent experiments is presented.

To confirm the native folding of wDIII and its proper display by the VLPs, the specific recognition of HBcAg-wDIII by the E16 mAb, was examined. E16 is a potent neutralizing mAb against wDIII and only binds to a conformational epitope that consists of four discontinuous secondary structural elements of the native wDIII [24]. If wDIII on HBcAg-wDIII VLPs can be recognized by E16, it would indicate its proper folding and display. Indeed, such specific and high affinity (Kd = 22.14 nM) binding of HBcAg-wDIII VLP to E16 was revealed by ELISA analysis (Figure 4). In contrast, no specific binding to an anti-Ebola IgG isotype control mAb (6D8) was detected (Figure 4). These results indicated that wDIII was displayed on the VLPs in its native conformation thereby suggesting the preservation of its WNV neutralization determinants.

### 3.4. HBcAg-wDIII VLPs Induced Potent Humoral Immune Response in Mice

**Figure 5:**
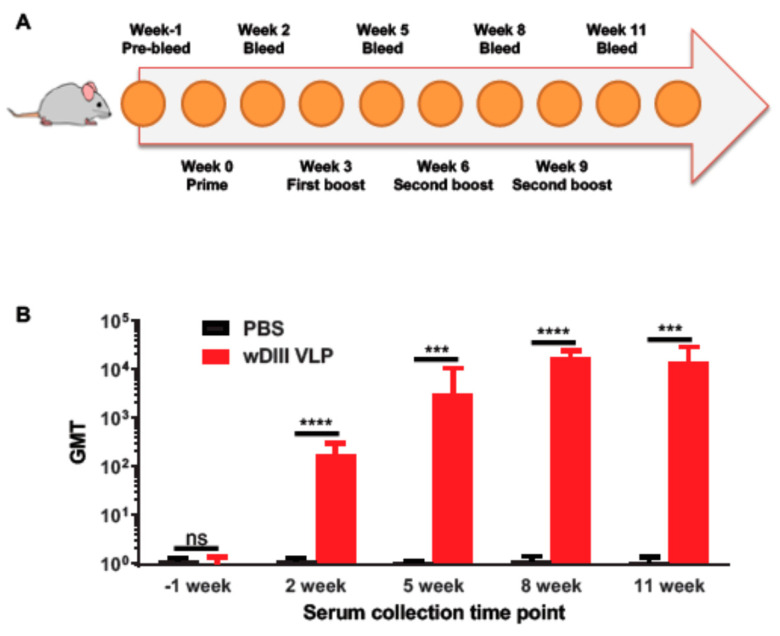
Antibody responses after subcutaneous immunization of HBcAg-wDIII. (**A**) Scheme of immunization. BALB/C mice were immunized on weeks 0, 3, 6, and 9. Blood samples were collected on the indicated weeks. (**B**) Antibody responses in sera of immunized mice. Serum wDIII-specific IgG was measured by ELISA. The y-axis shows the geometric mean titers (GMT) and the error bars show the 95% level of confidence of the mean. NS, ***, and **** indicate *p* values >0.05, <0.001, and <0.0001, respectively, of HBcAg-wDIII VLP-immunized serum compared to that of negative control serum from PBS-injected mice.

BALB/c mice were inoculated subcutaneously with four doses of 25 μg VLPs over an eight-week time period to evaluate the immunogenicity of plant-produced HBcAg-wDIII VLPs (Figure 5A). Mice in the negative group were injected with an equal volume of saline buffer (PBS). Serum wDIII-specific antibody responses from individual mice were measured by ELISA and the geometric mean titer (GMT) was calculated (Figure 5B). Sera collected from the PBS control group were negative for wDIII-specific IgG (titer < 10) regardless of the time points over which the samples were obtained during the course of immunization (Figure 5B). Negative results were also obtained for pre-immune sera for all groups collected prior to the first immunization (day 0) (Figure 5B). In contrast, injection of HBcAg-wDIII VLPs induced a potent wDIII-specific IgG response after the prime immunization (week 2, log titer > 2.3, *p* < 0.0001 of HBcAg-wDIII VLP-immunized serum compared with that of PBS) (Figure 5B). The IgG titer reached its peak two weeks after the second boost injection (week 8) (log titer > 4.3, *p* < 0.0001 of HBcAg-wDIII VLP-immunized serum compared with that of PBS) (Figure 5B). The fourth delivery of VLPs did not significantly further boost the wDIII-specific antibody response as the IgG titer at week 11 was similar to that of week 8 (*p* = 0.69, week 8 serum compared to week 11 serum) (Figure 5B). 

**Figure 6:**
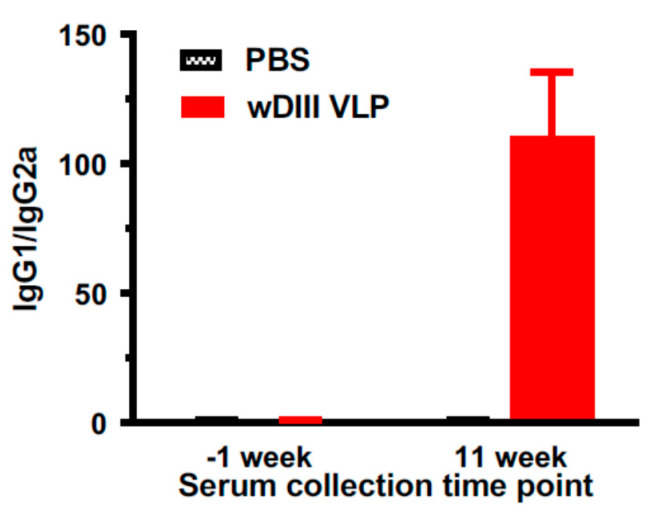
Anti-wDIII IgG subtypes elicited by immunization with HBcAg-wDIII VLPs. Sera collected at week −1 (before immunization) and week 11 from mice injected with HBcAg-wDIII VLP or PBS were measured for wDIII-specific IgG1 and IgG2a. The mean IgG1/IgG2a ratio and SD from three independent measurements were calculated for each mouse group.

ELISA was used to evaluate the Th type of response induced by HBcAg-wDIII VLPs. Analysis of sera collected at week 11 indicated that the wDIII-specific IgG subtype IgG1 titer was significantly higher than that of the IgG2a subtype (*p* = 0.0005) (Figure 6), indicating that a Th2-type biased response was stimulated by HBcAg-wDIII VLP with alum as the adjuvant. 

### 3.5. HBcAg-wDIII VLPs Elicited Antibodies that Bind to the Same Native Epitope as the Protective mAb E16

**Figure 7:**
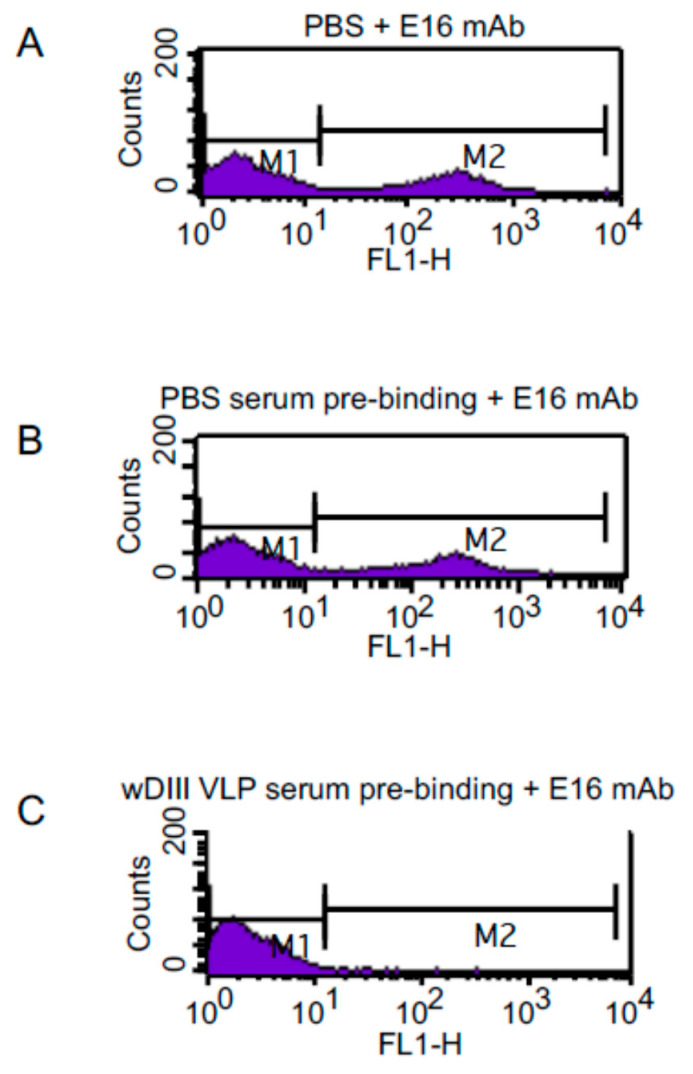
Competitive binding between the E16 mAb and antibodies in anti-HBcAg-wDIII serum to wDIII displayed on the yeast cell surface. wDIII-displaying yeast cells were pre-incubated with PBS (**A**) or week-11 pooled sera (1:1000 dilution) from mice either injected with PBS (**B**) or HBcAg-wDIII VLP (**C**). The E16 mAb was then incubated with yeast cells. The specific binding between E16 and yeast-displayed wDIII was measured by staining with an Alexa Fluor 488-conjugated goat anti-human secondary antibody and processing by flow cytometry.

We next investigated if HBcAg-wDIII VLP-induced antibodies could bind specifically to native wDIII epitopes and if some of these antibodies might even bind to the same epitope as mAbs that provide protection against lethal WNV infection. Week 11 antisera were evaluated for their ability in blocking the binding of E16, a protective anti-wDIII human IgG1 mAb, to yeast cells that surface-display wDIII in its native conformation. As expected, flow cytometric analysis demonstrated the specific binding between E16 and wDIII displayed on the surface of the yeast cells (Figure 7A). Pre-incubation of yeast cells with antisera from mice that were mock-immunized with PBS did not block such specific binding (Figure 7B). In contrast, pre-incubation with anti-HBcAg-wDIII VLP sera prevented E16 from binding to wDIII-displaying yeast cells (Figure 7C), suggesting that anti-HBcAg-wDIII VLP sera contained antibodies that competed with E16 in binding the same protective epitope. Yeast display experiments also confirmed the direct binding of antibodies in anti-HBcAg-wDIII VLP sera to wDIII displayed on yeast cells (not shown). This indicated that HBcAg-wDIII VLPs evoked the production of antibodies that recognize native wDIII epitopes and some of them might be potentially neutralizing and protective. 

## 4. Discussion

WNV has caused continuous outbreaks in the US since its introduction in 1999 and expanded into new territories in Europe and other parts of the globe with the risk of causing more frequent instances of neuroinvasive complications [2]. Therefore, it is urgent to develop a potent WNV vaccine to stop its geographic expansion and protect the public from this recurring epidemic. 

The wDIII has been produced in *E. coli*, insect cells, and plants in previous studies. Immunization of wDIII as a subunit vaccine with various adjuvants induced anti-WNV antibodies and even provided protection in mice in some instances [29,30,31,32,33]. These vaccine candidates, however, may not support their commercial production at the scale and/or cost to meet the demand for preventing global WNV epidemics. For example, the insoluble nature and presence of endotoxins in *E. coli*-produced wDIII preparations demand cumbersome and expensive processes of refolding and endotoxin removal, thereby hindering its ability to produce a large amount of vaccines at a low cost [29]. 

Our results indicated that wDIII can be produced rapidly in the form of HBcAg-wDIII VLPs in *N. benthamiana* plants using a transient plant expression system. Unlike insoluble aggregates produced in *E. coli*, HBcAg-wDIII VLPs produced in plants was soluble, allowing it to be directly purified to 95% homogeneity without the need for additional time-consuming refolding processes. The plant-produced HBcAg-wDIII VLPs were recognized by E16, a potent neutralizing mAb that binds to a conformational epitope on wDIII, but not the DIII of other flavivirus E proteins [34]. This supports our hypothesis that producing soluble wDIII in plants increases the likelihood of preserving its native epitopes, including potent neutralizing epitopes.

The high yield of HBcAg-wDIII VLPs in plants may help address the cost issue of vaccine production. Due to the need for building capital-intense cell culture facilities, bacterial and insect cell-based production of vaccines are often associated with limited scalability and high cost [35,36]. In contrast, plant-produced biomass and recombinant proteins can be generated at a large scale in inexpensive greenhouses with light, water, and fertilizer at a demonstrably lower cost [37,38,39,40]. The scalability of biomass production and downstream processing for transient plant expression, the platform used for HBcAg-wDIII VLPs expression in this study, has been demonstrated in both *N. benthamiana* and lettuce plants [41,42,43,44,45,46]. We previously produced wDIII by itself in plants and observed leaf necrosis, leading to a low yield (73 μg/g LFW) of wDIII [11]. Expression of wDIII in the form of HBcAg-wDIII VLP has overcome the issue of leaf necrosis and increased the yield to a high level of 1200 μg/g LFW, exceeding the yield requirement for vaccine manufacturing. This level of expression obtained under non-optimized laboratory conditions can even be further optimized using industrial-scale manufacturing growth conditions [47]. For downstream processing, HBcAg-wDIII VLPs were easily purified to homogeneity with a one-step process, which is routinely used for VLP vaccine production in the pharmaceutical industry and is in full compliance with regulations of current Good Manufacturing Practice (cGMP) [47]. Capsid protein-based VLPs including HBcAg-derived VLPs have been shown to have superior stability over other protein-based subunit vaccines under diverse physical stress conditions [48]. The combined advantages of rapid high-level production, facile purification, and increased stability of HBcAg-wDIII VLPs suggest the feasibility of using plants to produce HBcAg-wDIII VLP-based vaccines at a large scale and low cost. 

Our results showed that 25 μg of HBcAg-wDIII VLPs (equivalent to 7 μg wDIII antigen) induced a potent wDIII-specific humoral response with antigen-specific IgG titers (log titer) > 4.3 at week 8 and 11. This antibody response has the potential to be neutralizing and/or protective because (1) antibody responses to wDIII with similar IgG titers have been shown to be neutralizing and protect mice from lethal WNV challenges [29,49]; and (2) anti-HBcAg-wDIII VLP serum contained antibodies that competed with E16 at binding to the same protective epitope (Figure 7). While these results are promising, future neutralization and mouse protection experiments are required to verify if this vaccine candidate can actually provide protection against WNV. While HBcAg-wDIII VLPs evoked both IgG1 and IgG2a responses, IgG1 titers were higher than IgG2a, suggesting a Th2-biased response. This result is consistent with previous observations that flavivirus antigen with alum as adjuvant tends to induce Th2-biased type response [50,51]. Since alum has been approved as an adjuvant for human applications, the ability of HBcAg-wDIII VLPs with alum as the adjuvant in eliciting potent antibody response indicates its potential human application. 

The high degree of sequence homology between the E protein of WNV and that of related flaviviruses presents both opportunities and challenges for vaccine development. In general, vaccines that can induce cross-reactive immune responses among several related viruses are highly valued, as such broad-spectrum vaccines can be used to protect people from multiple viral pathogens. However, cross-reactive antigens are undesirable for flavivirus vaccine development due to the phenomenon of antibody-dependent enhancement of infection (ADE). ADE has been shown to be clinically relevant to dengue virus (DENV) infection [52]. Specifically, antibodies induced by one serotype of DENV during a primary infection can enhance the infection by another DENV serotype, leading to a potentially lethal shock syndrome in a secondary infection through ADE [53]. Mutual enhancement between WNV and zika virus infections has been observed [54]. Therefore, WNV vaccines that can induce cross-reactive antibodies would have the risk of augmenting infection by ADE-prone flaviviruses in vaccinated subjects [53]. Thus, a follow-up study is clearly warranted to investigate if the anti-wDIII response is WNV-specific or cross-reactive with related flaviviruses and if such immune response has any ADE activity.

## 5. Conclusions

In summary, we have demonstrated the rapid high-level production of HBcAg-wDIII VLP, its proper display of wDIII epitopes, effective purification, and its strong antigenicity that induced a potent humoral response with a potency equivalent to that of protective wDIII antigens in previous studies. These results indicate that plant-produced VLPs offer an attractive option for developing effective, safe, and low-cost vaccines against WNV and other infectious diseases.

## Data Availability

The data presented in this study are contained within this article.
